# Uniparental Inheritance of Chloroplast DNA Is Strict in the Isogamous Volvocalean *Gonium*

**DOI:** 10.1371/journal.pone.0019545

**Published:** 2011-04-29

**Authors:** Yuka Setohigashi, Takashi Hamaji, Mahoko Hayama, Ryo Matsuzaki, Hisayoshi Nozaki

**Affiliations:** Department of Biological Sciences, Graduate School of Science, University of Tokyo, Hongo, Bunkyo-ku, Tokyo, Japan; The Salk Institute, United States of America

## Abstract

**Background:**

A problem has remained unresolved regarding the exceptions to the unilateral inheritance of chloroplast DNA (cpDNA) from *MT+*/female in *Chlamydomonas* and other volvocaleans demonstrated by the previous genetic analyses. For identification of the parental types of cpDNA, these studies used parents that have differences in restriction fragment length polymorphisms and exhibit partial sexual incompatibility.

**Methodology/Principal Findings:**

In the present study, we used sexually compatible parents of the isogamous colonial volvocalean *Gonium maiaprilis* that seemed an ideal species to identify the pattern of cpDNA inheritance based on the length difference in the putative group I intron interrupted in the Rubisco large subunit gene and objective identification of mating types by the presence or absence of the minus-dominance (*MID*) gene. We examined patterns of inheritance of cpDNA and presence/absence of a *MID* ortholog (*GmMID*) in 107 F_1_ progeny of *G. maiaprilis* that were obtained by inducing germination of separated single zygotes. The results demonstrated no exception of the uniparental inheritance of cpDNA from the *MT+* parent (lacking *GmMID*) in sexually compatible or genetically less divergent strains of *G. maiaprilis*.

**Conclusions/Significance:**

The present data suggest that the uniparental inheritance of cpDNA is likely more strict in crossings of less diverged strains or sexually compatible parental volvocaleans, and some genetic inconsistency between the parents may cause exceptional uniparental inheritance of cpDNA.

## Introduction

Chloroplast DNA (cpDNA) in the volvocalean algae is predominantly transmitted from only one of the two parental mating types to the progeny; from mating type plus (*MT+*) in the isogamous species *Chlamydomonas reinhardtii* [1] and *Gonium pectorale* [2] or from female in the oogamous *Volvox carteri* [3]. However, these studies showed that 2–8% of the F_1_ progeny have an exceptional pattern of uniparental inheritance of cpDNA (cpDNA) [1]–[3], i.e. they inherit cpDNA from the *MT−*/male. For identification of the parental types of cpDNA, these studies used strains of complementary mating types (sexes) that have differences in restriction fragment length polymorphisms (RFLPs) and exhibit partial sexual incompatibility [2]–[4].

Studies of intra/interspecific crossings in mouse demonstrated that paternal mitochondrial DNA (mtDNA) is selectively eliminated during early embryogenesis in intraspecific crossings, whereas 50% of paternal mtDNA are transmitted to progeny in interspecifc crossings [5], [6]. Thus, crossings between pairs with partial sexual isolation or between genetically differentiated entities in the volvocaleans may also increase the exceptional rate of uniparental inheritance of organelle DNA when compared with intraspecific crossings.

**Figure 1:**
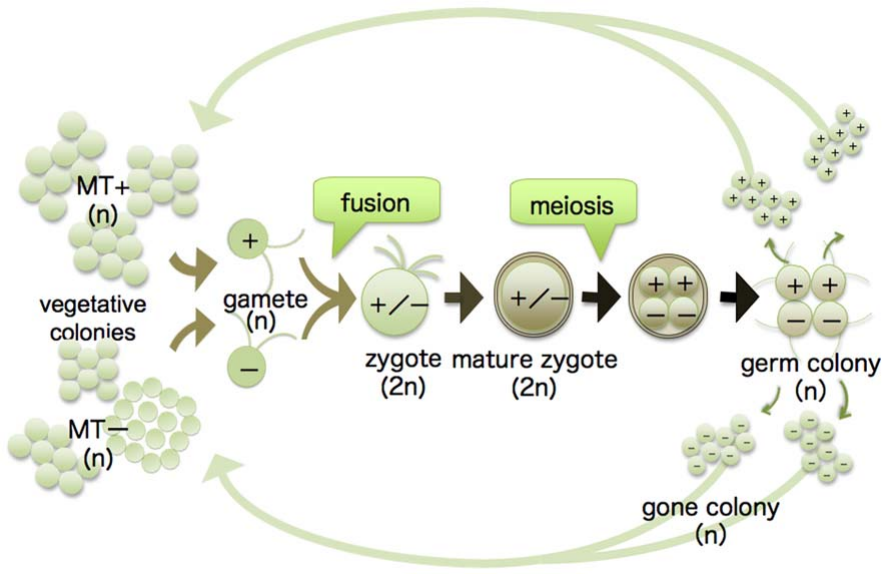
Diagram of sexual reproduction in heterothallic *Gonium maiaprilis*. Based on Hayama et al. [7].

*Gonium maiaprilis* is an isogamous colonial volvocalean that exhibits heterothallic sexuality [7] (Figure 1). The mating type (*MT−*)-determining minus dominance gene, *MID* [8], was recently identified in the closely related species *G. pectorale* [2]. In addition, our preliminary comparison of cpDNA sequences including a putative group I intron in the Rubisco large subunit (*rbcL*) genes indicated a difference in length of the introns among the *G. maiaprilis* strains. Thus, *G. maiaprilis* seems an ideal species to identify the pattern of cpDNA inheritance based on the difference in the group I intron and objective identification of mating types by the presence or absence of the *MID* gene [2].

In this study, we examined patterns of inheritance of cpDNA in 107 F_1_ progeny of *G. maiaprilis*. The results demonstrated no exception of the uniparental inheritance of cpDNA from the *MT+* parent in sexually compatible strains of *G. maiaprilis*.

## Results

One hundred and thirty-three gone colonies, each representing a separate meiotic product, were isolated from 44 germinating zygotes of *G. maiaprilis* Asa041901×Asa041903 to establish F_1_ strains (Figure 1). Ultimately, 77% (103/133) of the gone colonies became actively growing cultures. Based on backcrossing, 58 of the 103 exhibit a minus mating phenotype and the remaining 45 a plus mating phenotype (Table 1).

**Table 1:** Mating phenotypes, presence/absence of *GmMID* and inheritance of cpDNA in F_1_ progeny of *G. maiaprilis* Asa041901×Asa041903.

Mating phenotype^a^	No. of F_1_ strains	Presence of *GmMID* b	Absence of *GmMID* b	cpDNA from Asa041901(+)^b^	cpDNA from Asa041903(−)^b^
Mating type −	60	60	0	60	0
Mating type +	47	0	47	47	0
Total	107	60	47	107	0

a Based on backcrossing.

b Based on genomic PCR (Figures 2 and S4).

To determine the presence or absence of the *MID* gene, the *MID* orthologue (*GmMID*) was isolated from *G. maiaprilis* and characterized (Figures S1, S2, S3). Genomic PCR using *GmMID*-specific primers demonstrated that all 60 F_1_ strains (including additional two F_1_ strains previously established [7]) with minus mating phenotype have *GmMID* whereas all 47 F_1_ strains (including additional two F_1_ strains previously established [7]) with plus mating phenotype lack this gene (Figures 2 and S4). On the other hand, all 107 F_1_ strains had cpDNA of the Asa041901 (*MT+*) type based on genomic PCR using *rbcL* group I intron-specific primers (Figures 2, 3, S4 and Table 1).

**Figure 2:**
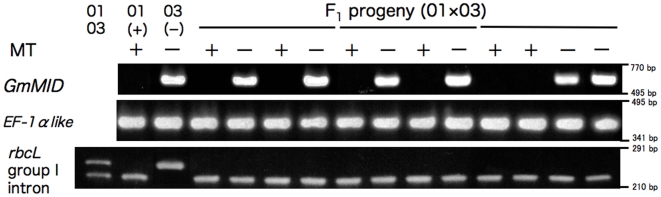
Mating phenotypes (MT) and results of genomic PCR for parental strains (Asa041901[01] and Asa041903 [03]) and 12 representative F_1_ strains of *Gonium maiaprilis*. Presence/absence of *GmMID* and the length polymorphism within the cpDNA*rbcL* group I intron are assessed by gel electrophoreses. The nuclear gene *EF-1alpha like* serves as a control. The horizontal line over the F_1_ progeny indicates F_1_ strains originating from the same zygote.

**Figure 3:**
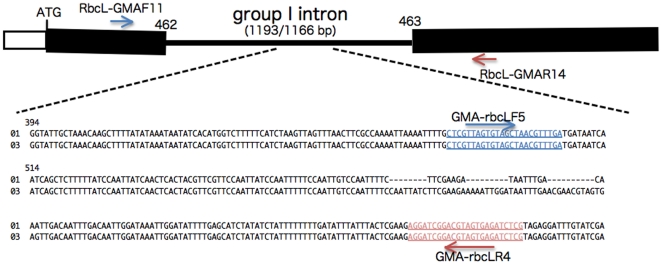
Diagram showing intron/exon structure and positions of specific primers (Table S2) in the *rbcL* genes from *Gonium maiaprilis* Asa041901 (01) and Asa041903 (03) (GenBank/EMBL/DDBJ accession nos. AB520743 and AB520744). Thick bars represent exons interrupted by a putative group I intron between basepairs 462 and 463 of the sequence of the *rbcL* gene of *Chlorella vulgaris* [accession no. AB001684]. Numbers above the alignment indicate the nucleotide position within the intron.

The secondary structures of the nuclear ribosomal DNA internal transcribed spaces 1 and 2 (ITS-1 and ITS-2) contain single base substitutions in four positions between *G. maiaprilis* Asa041901 and Asa041903 (Figure S5). These substitutions did not correspond to compensatory base change (CBC), suggesting that the strains fall within a range of an interfertile entity or a biological species [7], [9]. In *G. pectorale* Mongolia1 and Mongolia4, seven single base substitutions were detected in the ITS secondary structures (Table 2) although no CBC was recognized (Figure S6). Furthermore, the nucleotide sequences of the *rbcL* coding region (1128 bp) of the *G. maiaprilis* parents are exactly the same (GenBank/EMBL/DDBJ accession nos. AB520743-5, [7]) whereas one nucleotide substitution is present between the parents of *G. pectorale* (Table 2).

**Table 2:** Comparison of *Gonium maiaprilis* and *G. pectorale* crosses.

Source of zygotes		No. of nt change in ITS^a^ (*rbcL* b)	No. of F_1_ strains examined	Survival rate of F_1_ strains	cpDNA from *MT+* parent	cpDNA from *MT−* parent	Percentage of exceptions	Reference
Mating type +	Mating type −							
*G. pectorale* Mongolia1	*G. pectorale* Mongolia4	7 (1)	78	Poor	73	5	6.4%	Hamaji et al. [2]
*G. maiaprilis* Asa041901	*G. maiaprilis* Asa041903	4 (0)	107	78%	107	0	0%	This paper

a Total number in ITS-1 and ITS-2 of nuclear ribosomal DNA (GenBank/EMBL/DDBJ accession nos. AB520746 and AB623040-2) (Figures S5 and S6).

b Coding region of *rbcL* gene (1128 bp) (GenBank/EMBL/DDBJ accession nos. AB520743, AB520745, AB623038 and AB623039), in which *G. pectorale* sequences were determined in this study as described previously [7].

## Discussion

In 78 F_1_ strains of *G. pectorale* Mongolia1×Mongolia4, five exceptions of the uniparental inheritance of cpDNA were reported [2] (Table 2). In contrast, there were no exceptions of the uniparental inheritance of from the *MT+* parent (lacking *GmMID*) among the 107 *G. maiaprilis* F_1_ strains (Table 2 and Figure S4). This difference in the rate of exceptional uniparental inheritance of cpDNA between *G. maiaprilis* and *G. pectorale* is significant (P = 0.0014<0.05) by Fisher's exact test [10]. On the other hand, the survival rate of F_1_ progeny (77%) in *G. maiaprilis* is high as in intraspecific crossings of other volvocaleans (Table S1) [3], [11], [12]. In *G. pectorale* Mongolia1×Mongolia4, however, the survival of F_1_ progeny was poor thus obviating tetrad analysis [2]. In addition, genetic difference between *G. maiaprilis* Asa041901 and Asa041903 is smaller than that between *G. pectorale* Mongolia1 and Mongolia4 (Table 2). Therefore, reproductive/genetic isolation between *G. maiaprilis* Asa041901 and Asa041903 is apparently less than that between *G. pectorale* Mongolia1 and Mongolia4.

These results suggest that the uniparental inheritance of cpDNA may be more strict in crossings of less diverged strains or sexually compatible parental volvocalean parents, and some genetic inconsistency between the parents may cause exceptional uniparental inheritance of cpDNA. The difference in the rate of exceptional uniparental inheritance of cpDNA (Table 2) could be considered to result from the difference in maturation of zygotes prior to germination between G. *maiaprilis* and *G. pectorale*. The zygotes of G. *maiaprilis* were induce to germinate after six-week dark treatment while immature zygotes were used for germination in *G. pectorale* Mongolia1×Mongolia4 [2]. However, determination of the uniparental inheritance or complete digestion of cpDNA from *MT−* occurs in the early stage of zygote formation or quadriflagellate zygotes in *Chlamydomonas reinhardtii* [13]. Thus, the uniparental inheritance of cpDNA in the volvocaleans may be based on a precision molecular system that requires interactions of alleles from both parental cells, of sex-related genes that may be evolving rapidly [14], although details of the molecular mechanism for uniparental inheritance in the Volvocales remain unresolved [15].

Exceptional cases of the uniparental inheritance of mutations to streptomycin resistance in *Chlamydomonas reinhardtii* [16], [17] and the colonial volvocalean *Eudorina elegans* [18] were reported in classic genetic studies. However, these studies are based on crossings of UV-induced mutant strains that might have been affected by additional mutations causing confusion of the consortium of the parental cells for uniparental inheritance of the organelle DNAs.

## Materials and Methods

### Cultures and induction of sexual reproduction in *Gonium maiaprilis*

Two *G. maiaprilis* strains of complementary mating types (Asa041901 and Asa041903) were used in this study. These two strains are available from the Microbial Culture Collection at the National Institute for Environmental Studies (NIES-Collection [19]). The cultures were grown in screw-cap tubes (18×150 mm) containing about 10 mL VTAC or AF-6 medium modified by elimination of CaCO_3_ and addition of 400 mg L^−1^ MES [19]–[21]. Cultures were grown at 20°C, on a 14:10 h light-dark cycle, under cool-white fluorescent lamps at 165–175 µmol m^−2^ s^−1^ intensity.

For induction of sexual reproduction, approximately 10 ml of a 14-day-old culture in VTAC medium were reduced to 1 mL by centrifugation. The concentrated cultures of the two complementary mating types were mixed in Petri dishes (60-mm diameter) with 5.0 ml mating medium [20]. These dishes were cultured at 25°C on a 14:10 h light-dark cycle, under cool-white fluorescent lamps at 165–175 µmol m^−2^ s^−1^ intensity. After 10–14 days under these conditions, zygotes were pipetted onto the surface of AF-6 medium solidified with 1% agar in Petri dishes (90-mm diameter). The dishes were placed in the dark by wrapping in a double layer of aluminum foil and maintained in darkness at 20°C for about 6 weeks, after which the matured, clumped zygotes were separated using the pressure between a cover slip and slide. The separated zygotes were individually isolated and placed in 500 µL AF-6 liquid medium in a glass depression (20-mm diameter) in Petri dishes. In order to avoid evaporation from the medium containing a single zygote, 5 mL water solidified with 1% agar were placed in the bottom of the Petri dishes. The separated zygotes were then grown at 20°C on 14:10 h light-dark cycle. After the zygote had given rise to one to four gone colonies originating from a four-celled germ colon (Figure 1), each gone colony was transferred into a separate tube of AF-6 medium by a micropipette to establish an F_1_ strain. Mating phenotypes of F_1_ strains were determined by backcrossing.

### Isolation and characterization of *GmMID*

The cDNA sequence of *GmMID* (GenBank/EMBL/DDBJ accession no. AB623043)was obtained from *Gonium maiaprilis* Asa041903 (*MT−*) as described previously [2], [22]. The genomic sequence (GenBank/EMBL/DDBJ accession no. AB623044) was determined using *GmMID*-specific primers (Table S2) that were designed based on the cDNA sequence and using the methods described previously [22].

For phylogenetic analysis, 47 amino acids from the RWP-RK domain of GmMID (Figure S1) were aligned with five other MID proteins (Figure S2) and 25 RWP-RK domain-containing sequences from *Chlamydomonas reinhardtii* and *Volvox carteri* genome data [2], [23]. From this alignment, a maximum likelihood analysis using the WAG model [24] was conducted using RAxML [25] with a bootstrap analysis [26] based on 100 replicates. Bootstrap analyses of the maximum parsimony method (based on the full heuristic search with the tree bisection reconnection branch-swapping algorithm) and neighbor joining methods (using p-distances) were also carried out based on 1,000 replications, using PAUP 4.0b10 [27] and Clustal X [28], respectively.

### Determination of the length polymorphism within the cpDNA *rbcL* group I intron of *Gonium maiaprilis*

The nucleotide sequence of the putative *rbcL* group I intron from *G. maiaprilis* Asa041901 and Asa041903 (GenBank/EMBL/DDBJ accession nos. AB520743 and AB520744) was determined by direct sequencing [7] using two specific primers located in the adjoining *rbcL* coding regions (Table S2) and showed a 27 bp difference in sequence length between the two strains (Figure 3).

### Genomic PCR for parental and F_1_ strains of *Gonium maiaprilis*

Presence/absence of *GmMID* and the length polymorphism within the cpDNA *rbcL* group I intron are assessed by gel electrophoreses. The nuclear gene *EF-1alpha like* of *G. maiaprilis* (GenBank/EMBL/DDBJ accession nos. AB623051 and AB623052) was determined by direct sequencing [7] using specific primers (Table 2) and genomic DNA, and serves as a control. PCR was performed with two specific primers for each gene (Table S2) and TaKaRa LA Taq (Takara bio inc., Shiga, Japan), under the following conditions: 30 cycles of 94°C for 30 seconds, 55°C for 30 seconds, and 72°C for 40 seconds, followed by 72°C for 7 minutes.

### ITS-1 and ITS-2 secondary structures

The ITS-1 and ITS-2 sequences (GenBank/EMBL/DDBJ accession nos. AB623040-2) were directly determined by the methods described in Hayama et al. [7] with primers for ITS regions (Table S2). The secondary structures of ITS-1 and ITS-2 were predicted using CentroidFold [29], [30] and revise the secondary structure models of ITS-1 and ITS-2 from earlier studies [7], [9], [31]–[33].

## Supporting Information

Figure S1 **Comparison of exon-intron structure between** ***GmMID*** **and five other** ***MID*** **homologs.**
(TIF)

Figure S2 **Alignment of six MID proteins from** ***Volvox carteri*** **(VcMID),** ***Pleodorina starrii*** **(PlestMID),** ***Gonium pectorale*** **(GpMID),** ***G. maiaprilis*** **(GmMID),** ***Chlamydomonas reinhardtii*** **(CrMID), and** ***C. globosa*** **(previously misidentified as** ***C. incersta*** **** [**34**] **) (CiMID).** Solid and shaded backgrounds indicate identity in 100% or in over 60% of the sequences aligned, respectively. Five amino acids composing a leucine zipper are marked with asterisks. A line above the alignment marks the RWP-RK domain of 47 amino acids used for the phylogenetic analyses (Figure S3).
(TIF)

Figure S3 **Maximum likelihood (ML) phylogenetic tree showing MID proteins from** ***Volvox carteri*** **(VcMID),** ***Pleodorina starrii*** **(PlestMID),** ***Gonium maiaprilis*** **(GmMID),** ***G. pectorale*** **(GpMID),** ***Chlamydomonas reinhardtii*** **(CrMID) and** ***C. globosa*** **(previously misidentified as** ***C. incersta*** **** [**34**] **) (CiMID).** Other members of the RWP-RK family from *Chlamydomonas* and *Volvox* are included as outgroup. Numbers next to branch points are bootstrap values for ML/neighbor joining/maximum parsimony methods.
(TIF)

Figure S4 **Summary of mating phenotypes (MT), presence (gray)/absence (white) of** ***GmMID*** **and types of cpDNA (** ***rbcL*** **group I intron) from parental strains (Asa041901[01] and Asao41903 [03]) and their 107 F_1_ strains of** ***Gonium maiaprilis*** **. White or gray box represents the same character as that of Asa041901 or Asa041903, respectively, for each of the three attributes.** Each horizontal line indicates those F_1_ strains originating from the same germinating zygote. Isolation of progeny representing both mating types in the 3 and 4-membered tetrads indicate that these are meiotic products.
(TIF)

Figure S5 **Secondary structures of the ITS-1 and ITS-2 RNA transcript of** ***Gonium maiaprilis*** **Asa041901 and Asa041902 (GenBank/EMBL/DDBJ accession nos. AB520746 and AB623042).** Arrows mark the four single base substitutions between Asa041901 and Asa041903. The number between the two characters indicates the nucleotide position where the single base substitution occurred; the left character is the base of Asa041901 whereas the right character is the base of Asa041903.
(TIF)

Figure S6 **Secondary structures of the ITS-1 and ITS-2 RNA transcript of** ***Gonium pectorale*** **Mongolia1 and Mongolia4 (GenBank/EMBL/DDBJ accession nos. AB623040 and AB623041).** Arrows mark the seven single base substitutions between Mongolia1 and Mongolia4. The number between the two characters indicates the nucleotide position where the single base substitution occurred; the left character is the base of Mongolia1 whereas the right character is the base of Mongolia4.
(TIF)

Table S1 Survival rates of F_1_ progeny from intra and interspecific crossing in various colonial volvocaceans.
(DOC)

Table S2 Primers used for amplifications and sequencing of four DNA regions in the present study.
(DOC)
