# Supplementary figures and images for: Uniparental Inheritance of Chloroplast DNA Is Strict in the Isogamous Volvocalean Gonium
Source: PLoS One. 2011 Apr 29;6(4):e19545. doi: 10.1371/journal.pone.0019545 (PMC3085477; doi:10.1371/journal.pone.0019545)

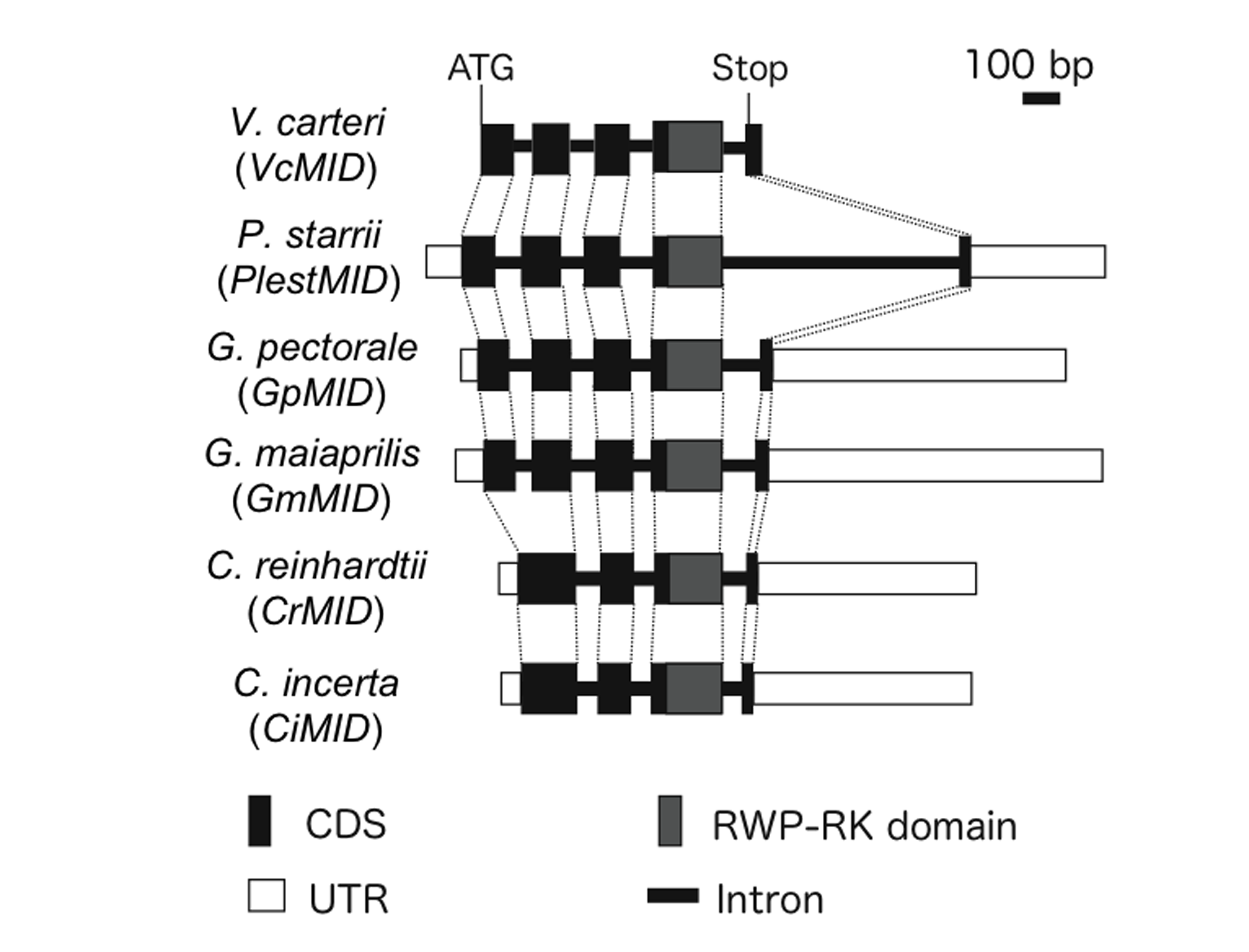

Supplement: Figure S1 — Comparison of exon-intron structure between GmMID and five other MID homologs. (TIF) [file pone.0019545.s001.tif]

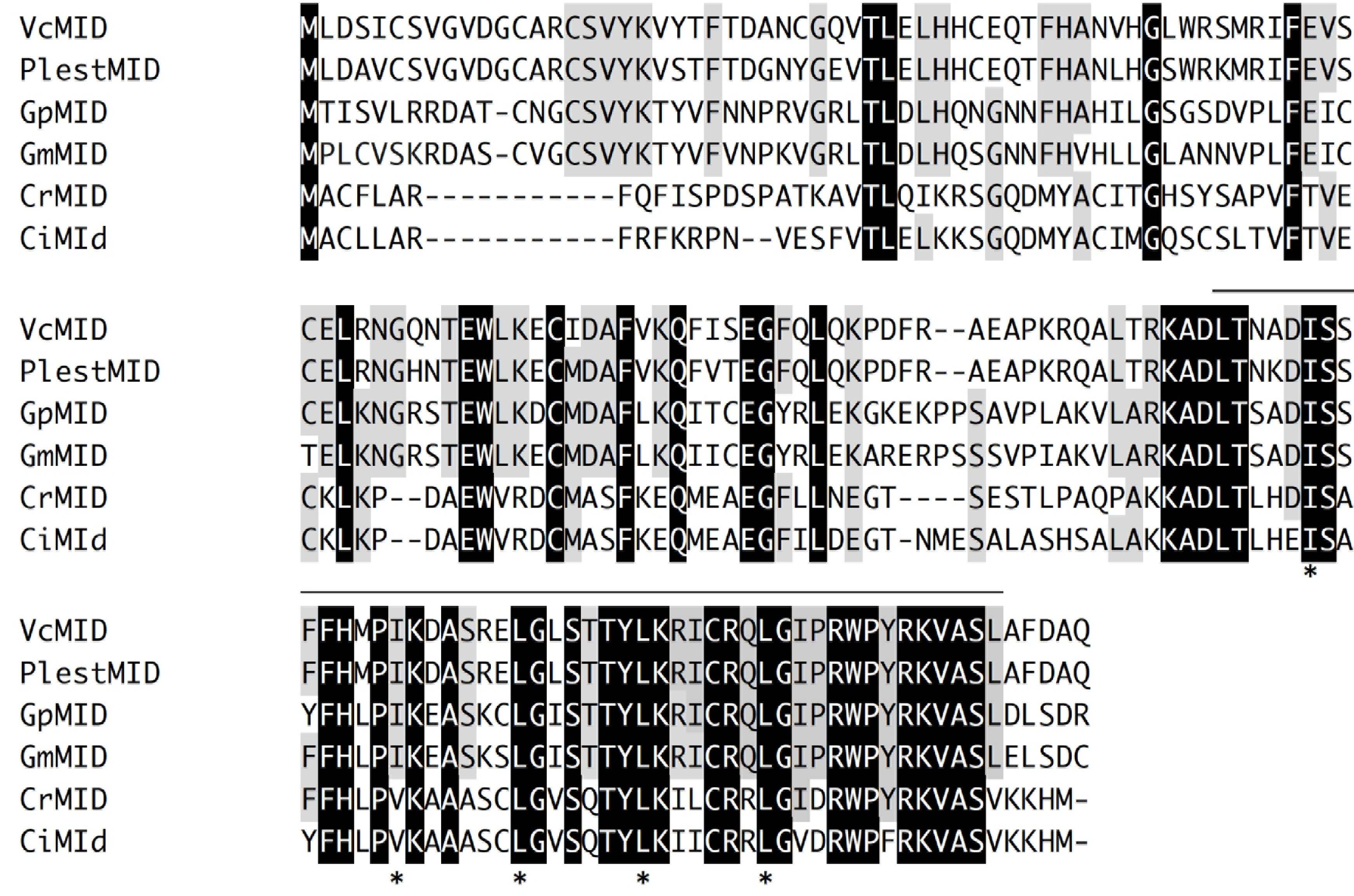

Supplement: Figure S2 — Alignment of six MID proteins from Volvox carteri (VcMID), Pleodorina starrii (PlestMID), Gonium pectorale (GpMID), G. maiaprilis (GmMID), Chlamydomonas reinhardtii (CrMID), and C. globosa (previously misidentified as C. incersta [34] ) (CiMID). Solid and shaded backgrounds indicate identity in 100% or in over 60% of the sequences aligned, respectively. Five amino acids composing a leucine zipper are marked with asterisks. A line above the alignment marks the RWP-RK domain of 47 amino acids used for the phylogenetic analyses (Figure S3). (TIF) [file pone.0019545.s002.tif]

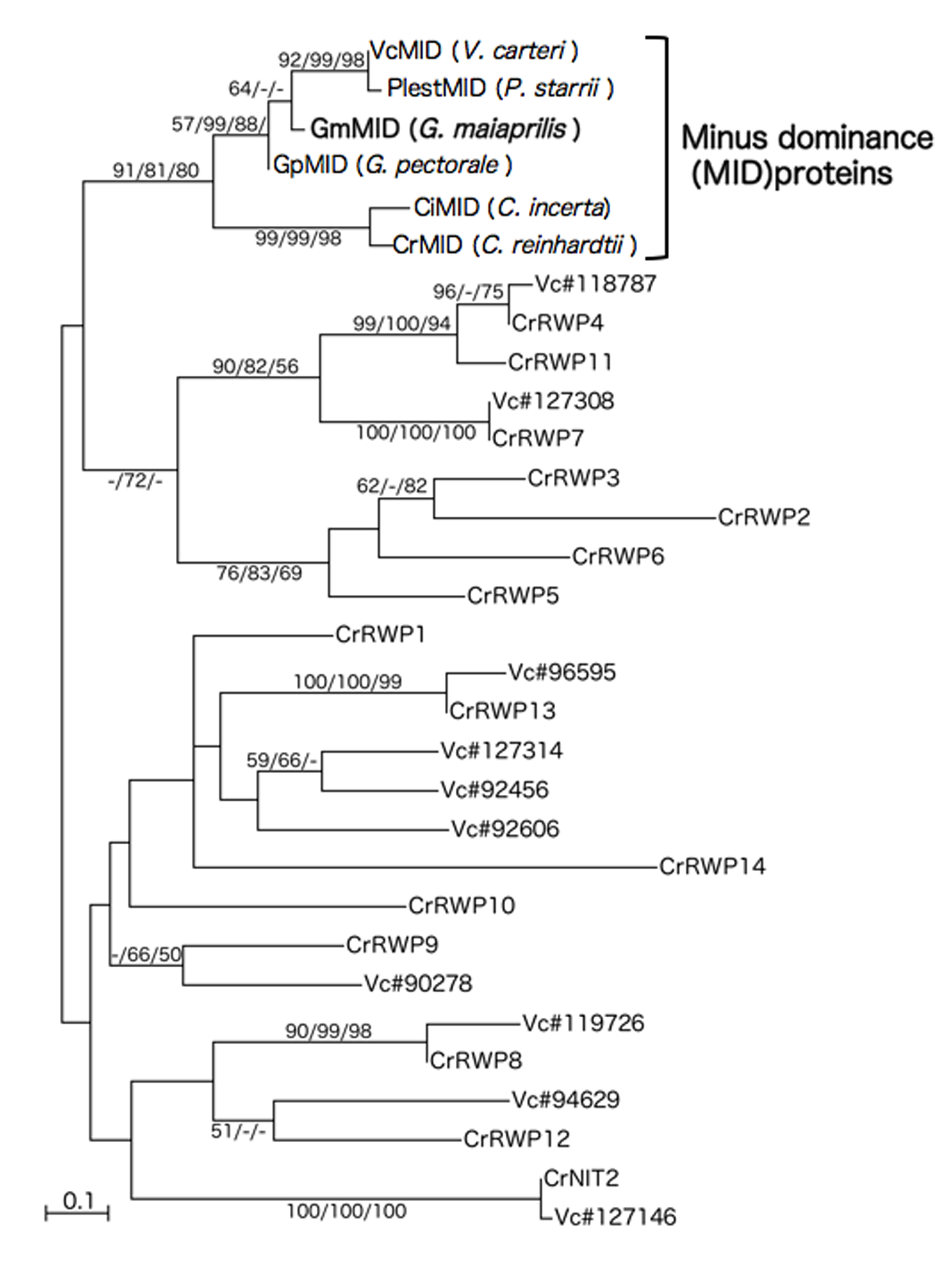

Supplement: Figure S3 — Maximum likelihood (ML) phylogenetic tree showing MID proteins from Volvox carteri (VcMID), Pleodorina starrii (PlestMID), Gonium maiaprilis (GmMID), G. pectorale (GpMID), Chlamydomonas reinhardtii (CrMID) and C. globosa (previously misidentified as C. incersta [34] ) (CiMID). Other members of the RWP-RK family from Chlamydomonas and Volvox are included as outgroup. Numbers next to branch points are bootstrap values for ML/neighbor joining/maximum parsimony methods. (TIF) [file pone.0019545.s003.tif]

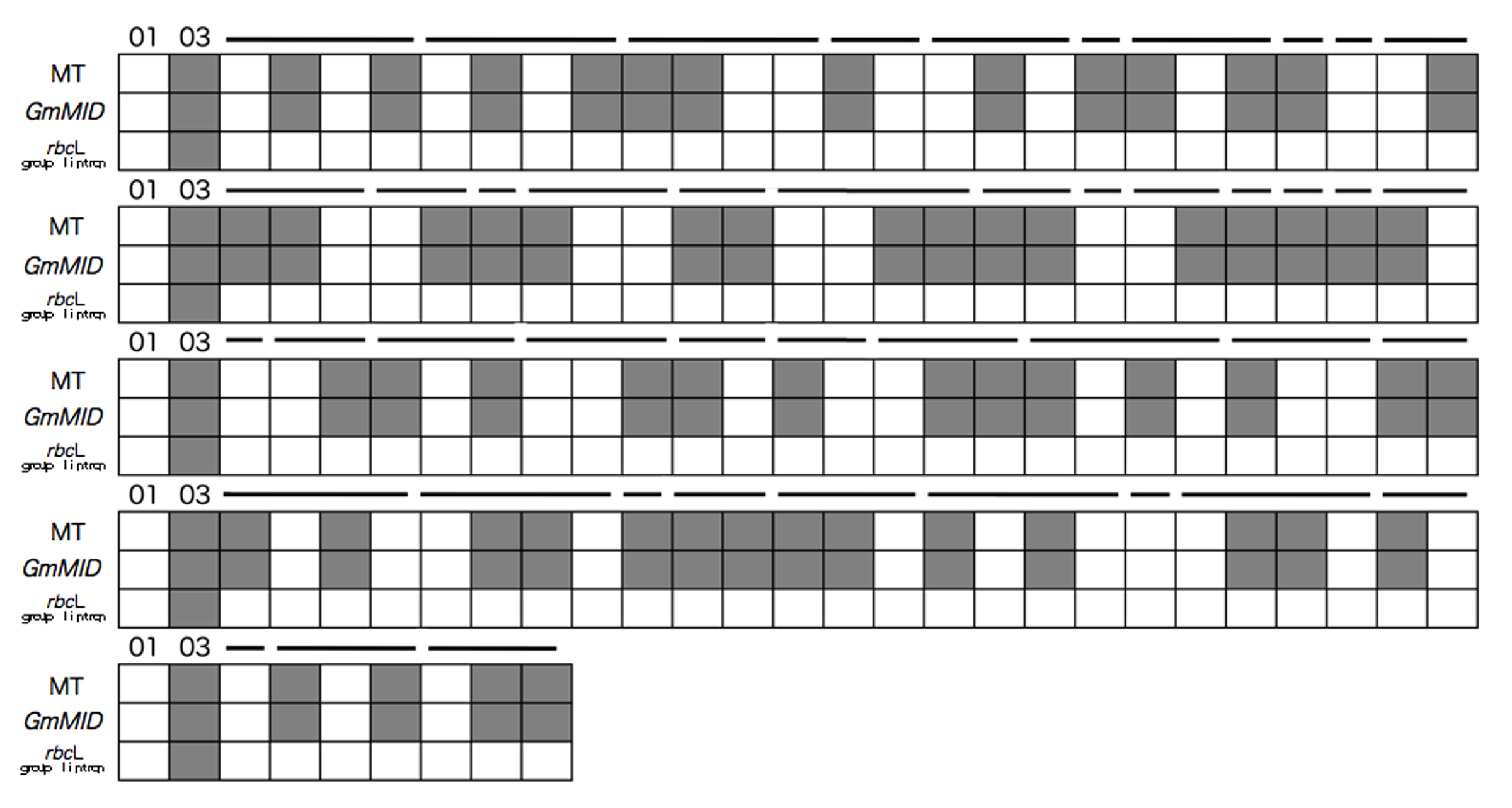

Supplement: Figure S4 — Summary of mating phenotypes (MT), presence (gray)/absence (white) of GmMID and types of cpDNA ( rbcL group I intron) from parental strains (Asa041901[01] and Asao41903 [03]) and their 107 F1 strains of Gonium maiaprilis . White or gray box represents the same character as that of Asa041901 or Asa041903, respectively, for each of the three attributes. Each horizontal line indicates those F1 strains originating from the same germinating zygote. Isolation of progeny representing both mating types in the 3 and 4-membered tetrads indicate that these are meiotic products. (TIF) [file pone.0019545.s004.tif]

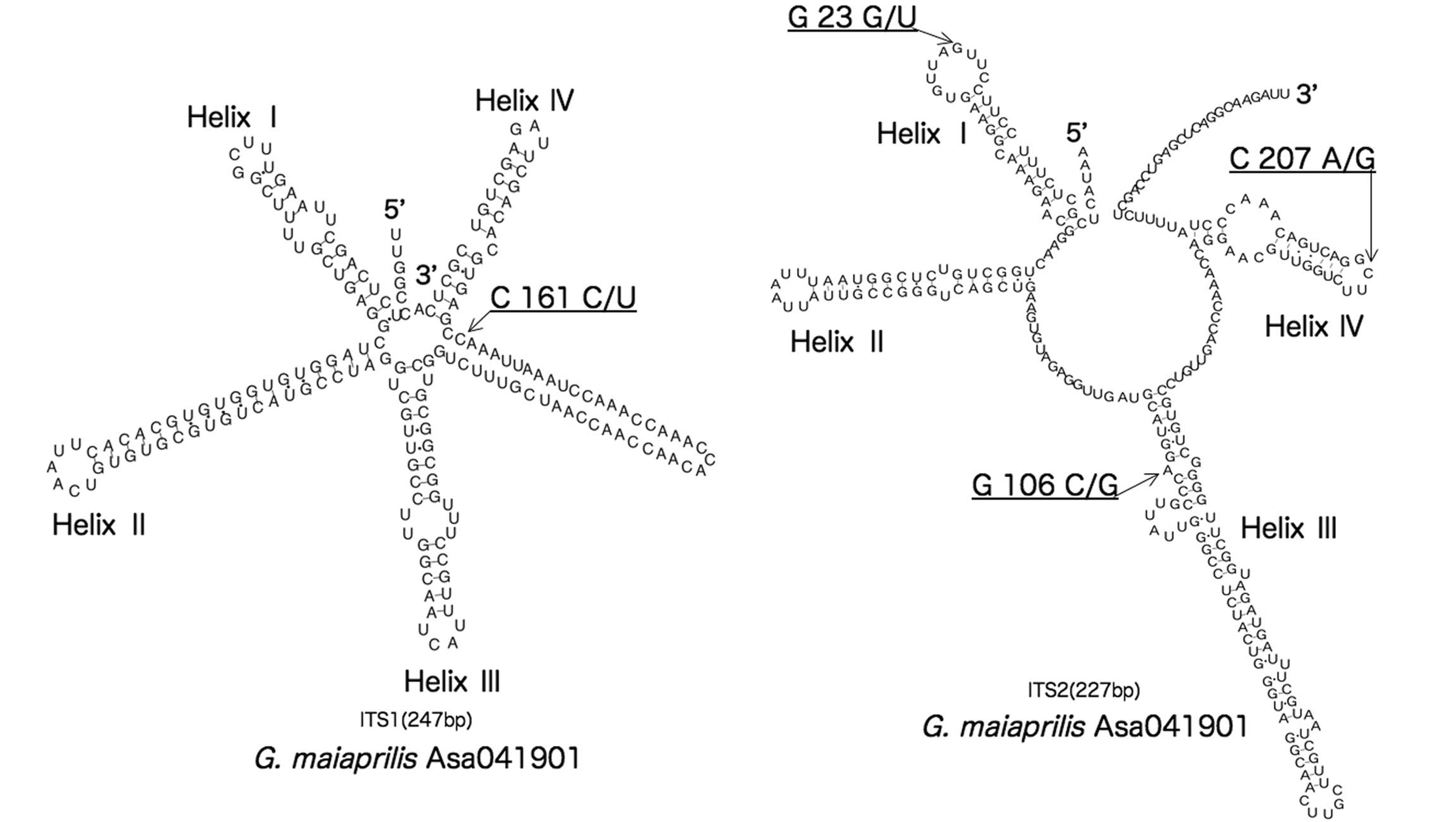

Supplement: Figure S5 — Secondary structures of the ITS-1 and ITS-2 RNA transcript of Gonium maiaprilis Asa041901 and Asa041902 (GenBank/EMBL/DDBJ accession nos. AB520746 and AB623042). Arrows mark the four single base substitutions between Asa041901 and Asa041903. The number between the two characters indicates the nucleotide position where the single base substitution occurred; the left character is the base of Asa041901 whereas the right character is the base of Asa041903. (TIF) [file pone.0019545.s005.tif]

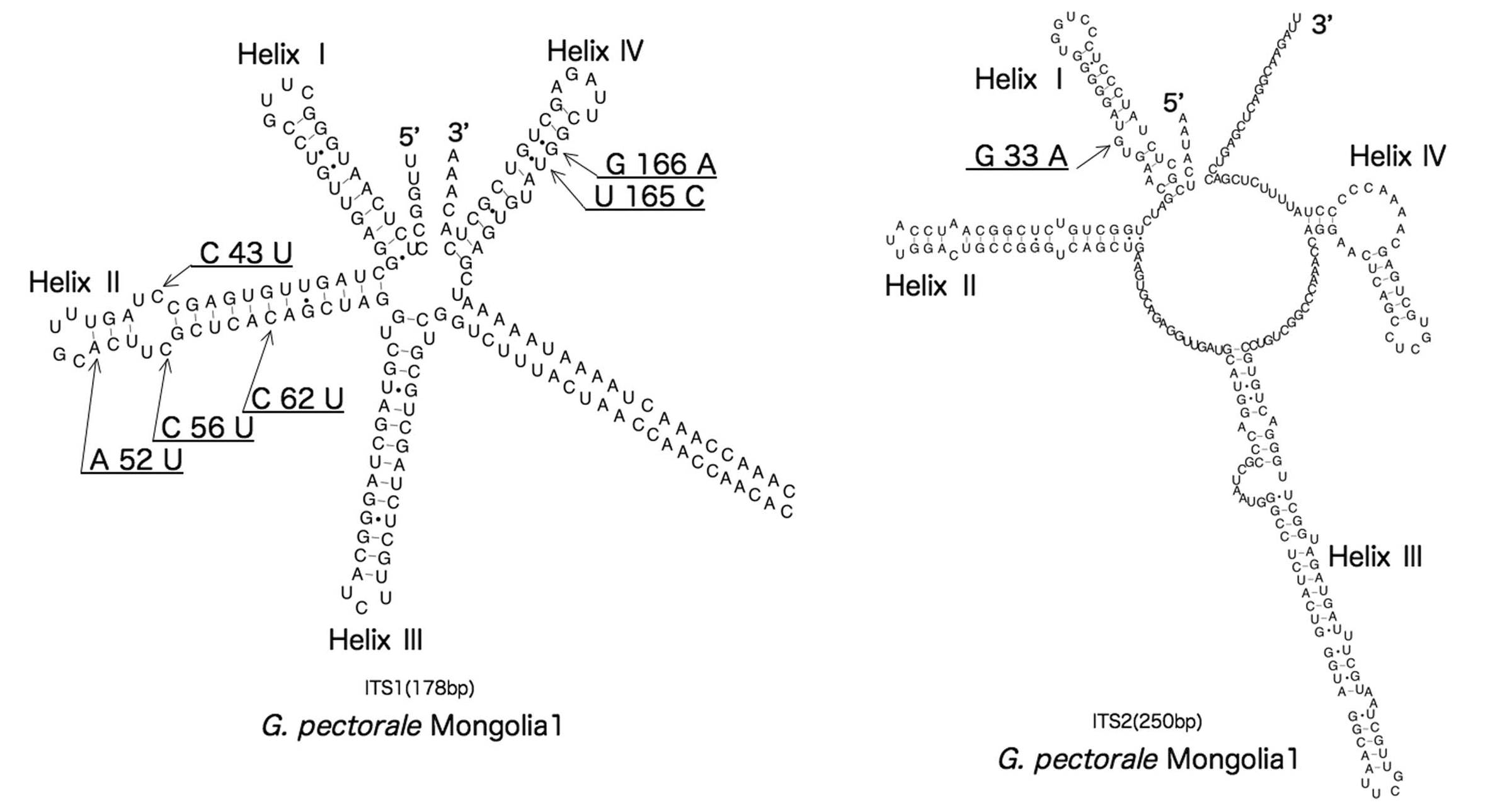

Supplement: Figure S6 — Secondary structures of the ITS-1 and ITS-2 RNA transcript of Gonium pectorale Mongolia1 and Mongolia4 (GenBank/EMBL/DDBJ accession nos. AB623040 and AB623041). Arrows mark the seven single base substitutions between Mongolia1 and Mongolia4. The number between the two characters indicates the nucleotide position where the single base substitution occurred; the left character is the base of Mongolia1 whereas the right character is the base of Mongolia4. (TIF) [file pone.0019545.s006.tif]
