# Supplementary material for: Uniparental Inheritance of Chloroplast DNA Is Strict in the Isogamous Volvocalean Gonium
Source: PLoS One. 2011 Apr 29;6(4):e19545. doi: 10.1371/journal.pone.0019545 (PMC3085477; doi:10.1371/journal.pone.0019545)
Supplement: Table S1 — Survival rates of F1 progeny from intra and interspecific crossing in various colonial volvocaceans. (DOC) [file pone.0019545.s007.doc]

**Table S1. Survival rates of F_1_ progeny from intra and interspecific crossing in various colonial volvocaceans**

| Source of Zygotes | | | | | inter/intra specific cross | | No. of gone colonies obtained | No. of surviving gone colonies | Survival rate of F_1_(gone colonies) | Reference |
| --- | --- | --- | --- | --- | --- | --- | --- | --- | --- | --- |
|  | Mating type+/Female parent | | Mating type−/Male parent | |
| *Volvox carteri* | | | |  |  | |  |  |  |  |
|  | f. *kawasakiensis* | | f. *nagariensis* | | inter | | 8 ^a^ | 0 | 0% | Nozaki (1988) ^b^ |
|  | f. *nagariensis* (Japan) | | f. *nagariensis* (Japan) | | intra | | 457 | 425 | 93% | Adams *et al.* (1990) ^c^ |
|  | f. *nagariensis* (India) | | f. *nagariensis* (India) | | intra | | 415 | 378 | 91% | Adams *et al.* (1990) |
|  | f. *nagariensis* (Japan) | | f. *nagariensis* (India) | | inter | | 632 | 79 | 13% | Adams *et al.* (1990) |
|  | f. *nagariensis* (India) | | f. *nagariensis* (Japan) | | inter | | 462 | 56 | 12% | Adams *et al.* (1990) |
| *Eudorina* | |  | | |  | |  |  |  |  |
|  | *E. elegans* | | *E. elegans* | | intra | | 100~500 | - | 69~97 % | Goldstein (1964) ^d^ |
|  | *E. illinoisensis* | | *E. illinoisensis* | | intra | | 200 | - | 91% | Goldstein (1964) |
|  | *E. elegans* | | *E. illinoisensis* | | inter | | 208 | - | 24% | Goldstein (1964) |
|  | *E. illinoisensis* | | *E. elegans* | | inter | | 166 | - | 33% | Goldstein (1964) |
| *Gonium* | | | |  |  | |  |  |  |  |
|  | *G. maiaprilis* Asa041901 | | | *G. maiaprilis* Asa041903 | |  | 137 | 107 | 78% | This paper |

^a^ Gone cells obtained.

^b^ Nozaki H (1988) Phycologia 27: 209-220.

^c^ Adams et al. (1990) Curr Genet 18: 141–153.

^c^ Goldstein (1964) J Protozool 11: 317-344.
