# Supplementary material for: Uniparental Inheritance of Chloroplast DNA Is Strict in the Isogamous Volvocalean Gonium
Source: PLoS One. 2011 Apr 29;6(4):e19545. doi: 10.1371/journal.pone.0019545 (PMC3085477; doi:10.1371/journal.pone.0019545)
Supplement: Table S2 — Primers used for amplifications and sequencing of four DNA regions in the present study. (DOC) [file pone.0019545.s008.doc]

**Table S2. Primers used for amplifications and sequencing of four DNA regions in the present study**

| Region | Designation | Sequence (5' to3') |
| --- | --- | --- |
| *MID* | MID-GMAF1 ^a^ | GCAGGCAATTGGGCATACCTCGCTG |
|  | MID-GMAR2 ^a, b^ | CTTATACACTGAGCAACCCACGCAT |
| *rbcL* ^c^ | RbcL-GMAF11 | GAAGAAGGCTCTGTAACAAACATG |
|  | RbcL-GMAR14 ^b^ | CACTCATAAACAGCACGACCATAG |
|  | GMA-rbcLF5 ^a^ | CTCGTTAGTGTAGCTAACGTTTGA |
|  | GMA-rbcLR4 ^a, b^ | CGAGATCTCACTACGTCCGATCCT |
| *EF-1α like* | CV_EF1A2-F1 ^d^ | GAGCGTGAGCTGGAGAAGCTGAAGG |
|  | CV_EF1A2-R2 ^b, d^ | ACACGCTTGTGGTGCATCTCCA |
|  | GMA_EF1A_F2 ^a^ | TGGTTCCCGCCGACGGTAACTTCA |
|  | GMA_EF1A_R2 ^a, b^ | ATCTTGTTCACACCGACGATCAGC |
| ITS | ITS-a ^e^ | GGGATCCGTTTCCGTAGGTGAACCTGC |
|  | ITS-b ^b,e^ | GGGATCCATATGCTTAAGTTCAGCGGGT |

^a^ Primer used for genomic PCR (Figure 2).

^b^ Reverse primer.

^c^ See Figure 3.

^d^ Designed based on *Chlamydomonas reinhardtii EFA2* sequence (Genbank/EMBL/DDBJ accession no. XM_001696516)

^e^ Coleman *et al.* (1994) Protist 149: 135–146..
